# Biodiversity and Temporal Distribution of Immature Culicidae in the Atlantic Forest, Rio de Janeiro State, Brazil

**DOI:** 10.1371/journal.pone.0159240

**Published:** 2016-07-12

**Authors:** Jeronimo Alencar, Cecília Ferreira de Mello, Nicolau Maués Serra-Freire, Anthony Érico Guimarães, Hélcio R. Gil-Santana, Raquel M. Gleiser

**Affiliations:** 1 Diptera Laboratory, Oswaldo Cruz Institute (Fiocruz), Rio de Janeiro, Brazil; 2 Postgraduate Program on Animal Biology, Institute of Biology, Federal Rural University of Rio de Janeiro, Seropédica, RJ, Brazil; 3 National Reference Laboratory on Rickettsioses Vectors, Oswaldo Cruz Institute (Fiocruz), Rio de Janeiro, Brazil; 4 Centro de Relevamiento y Evaluación de Recursos Agrícolas y Naturales-Instituto Multidisciplinario de Biología Vegetal (Consejo Nacional de Investigaciones Científicas y Técnicas—Universidad Nacional de Córdoba, CONICET-UNC), Facultad de Ciencias Exactas, Físicas y Naturales, Universidad Nacional de Córdoba, Córdoba, Argentina; Cary Institute of Ecosystem Studies, UNITED STATES

## Abstract

To increase the knowledge of biodiversity and identify larval habitats used by immature mosquitoes in the Atlantic Forest, we conducted a study in areas with various stages of preservation within the Guapiaçu Ecological Reserve in Cachoeiras de Macacu, Rio de Janeiro state. The Culicidae fauna were sampled during February, April, June, August, October, and December 2012; February, March, April, May, June, August, October, and December 2013; and January and March 2014. Immature mosquitoes were collected with dippers and suction tubes (mouth aspirators). Over the sampling period, 2697 larvae of 56 species were collected, some of which are recognized vectors of human diseases. The larval mosquito community found in artificial habitats, temporary ground water, and phytotelmata differed between sites, except for the mosquito fauna in bromeliads, which were almost 80% similar. Species segregation was more evident between larval habitats than between sites. *Culex usquatus* was the dominant species and colonized the highest number of larval habitats. The artificial larval habitats found in REGUA were colonized by a great diversity of species and high abundance as well, thus human artifacts left by the public in the area that collect water may promote an increase in mosquito populations. Among the species collected, some are known or suspected vectors of pathogens to humans and/or veterinary relevance, and their medical relevance is discussed.

## Introduction

Recent estimates suggest that there are 3 to 6 million arthropod species on Earth [1–3] (Thomas, 1990; Ødegaard, 2000; Hamilton et al. 2010), while no more than 30% of tropical insects have been described to date [4] (Godfray et al. 1999). Tropical forests harbor most of the insect diversity in the world [5] (Lamarre et al. 2012), but also account for the largest gaps in faunal knowledge [6] (Rapini et al. 2006). There is an urgent need to increase wildlife inventories, including surveys of insect fauna, in countries like Brazil, which are mainly tropical and have high biodiversity. Such countries are currently facing alarming habitat destruction, with species becoming extinct before they can be discovered or described by scientists [6] (Rapini et al. 2006). In Brazil, for instance, for the 40 year period between 1975 and 2014, an average yearly net forest cover change rate of -0.71% was estimated (Velasco Gomez et al. 2015). Between 1990 and 2010, based on high resolution satellite imagery, it was estimated that South and Central America and the Caribbean lost 56.9 million ha of its forest area (Achard et al. 2014).

Mosquitoes, which comprise the family Culicidae, are nematoceran dipterans of cosmopolitan distribution. In medical entomology, they have certainly attracted much attention from the public health sector, because they are involved in pathogen transmission to humans and domestic animals [7] (Forattini, 2002). There are 3549 recognized mosquito species distributed in approximately 112 genera [8] (Harbach 2008) or 42 genera according to the more traditional classification of Wilkerson et al. (2015) [9]. The neotropical region holds the highest level of endemicity, as 27% of the species are restricted to this biogeographical region [10] (Ward, 1982).

Knowledge of the distribution and abundance of mosquitoes in primary Atlantic Forest remnants typical of Brazil is of great importance for understanding the eco-epidemiology of mosquito-borne diseases. The Atlantic Forest biome has a high diversity of flora and vertebrate and invertebrate fauna, allowing a multiplicity of niche options for the development of culicids. Although research on ecological aspects of the Culicidae fauna has been undertaken in several areas of Brazil, in Rio de Janeiro (RJ) state, studies have focused on the presence of infectious agent vectors, mostly in the urban environment [11] (Alencar et al. 2011). In fact, most studies involving mosquitoes are developed due to the occurrence of a disease or the presence of its vectors in risk areas [12] (Guimarães & Arlé, 1984). On the other hand, research on the diversity of mosquitoes in their natural environment may reveal unknown habits of these vectors [13] (Hutchings et al. 2005).

A connection between species diversity and transmission of vector-borne diseases has long being suspected and there are several examples that community diversity can have contrasting effects on disease risk (Keesing et al. 2006, Johnson and Thieltges 2010). A diversified community offers great opportunity of adaptation of newly introduced or emerged viruses to suitable vectors and reservoirs. The dilution effect hypothesis refers to the phenomenon when increased species diversity reduces risk of disease, through different mechanisms such as deflecting transmission events from highly competent to less competent hosts (see Keesing et al. 2006 for discussion on several mechanisms underlying the effects of diversity). Alternatively, increased species diversity can increase risk (“amplification effect”), for example when increasing vector numbers. Nevertheless, there is growing evidence that the occurrence of dilution or amplification depends more on specific community composition than on biodiversity *per se* (Randolph and Dobson 2012), highlighting the importance of knowledge of culicid composition and ecology.

To contribute to the knowledge of the culicid fauna of the Atlantic Forest, and particularly of the Guapiaçu Ecological Reserve in the Municipality of Cachoeiras de Macacu, Brazil, the objectives of this study were to identify and compare the immature Culicidae fauna in different collection sites in terms of species richness, abundance and composition and to determine the ecological indicators for the species.

## Materials and Methods

### Ethics statement

All research was performed in accordance with scientific license number 34911 provided by SISBIO/IBAMA (Authorization and Information System on Biodiversity/ Brazilian Institute of Environment and Renewable Natural Resources) for the capture of culicids throughout the Brazilian national territory. The sampling in the area studied was also authorized by its owners, Nicholas and Rachel Locke, and the research coordinator Jorge Bizarro.

### Study area and mosquito sampling

Sampling was performed in the Guapiaçu Ecological Reserve (REGUA), located in the Municipality of Cachoeiras de Macacu, RJ, a 6500 ha conservation area created in 1996. According to the classification of Veloso et al. (1991) [14], REGUA’s plant cover is characterized as dense rain forest, varying in three different plant physiognomies. Descriptions of the plant cover and soils of the study area are provided in Alencar et al. (2015) [15]. Briefly, in the lower parts of the studied landscape, the original forest formation is dense alluvial rain forest, located in a flat terrain area, with pasture vegetation types and 7 years of reforestation. In areas of coastal plains, covering gentle slopes, the original forest was characterized as lowlands dense rain forest, where 3 and 5 year reforestations are now found. The highest portion of the studied landscape was the submontane topographical range, contemplating hilly and rugged relief, with forest cover characterized as submontane and montane dense rain forest. This area of mature forest is considered a control area for reforestation studies.

According to Köppen (1936) [16], the climate is tropical with rainy summers and dry winters, classified as type Af (Tropical rainforest climate). The average annual temperature is 22.4°C, with the maximum in January and February and the minimum in June. The average annual rainfall is 2095 mm, with December and January being the rainiest months and June and July the least rainy (Couto, 2010) [17].

Samples were obtained in the following months: February, April, June, August, October, and December 2012; February, March, April, May, June, August, October, and December 2013; and January and March 2014. Each month, the collections were performed on two consecutive days, between 8:00 am and 5:00 PM, in two areas of REGUA.

**Fig 1:**
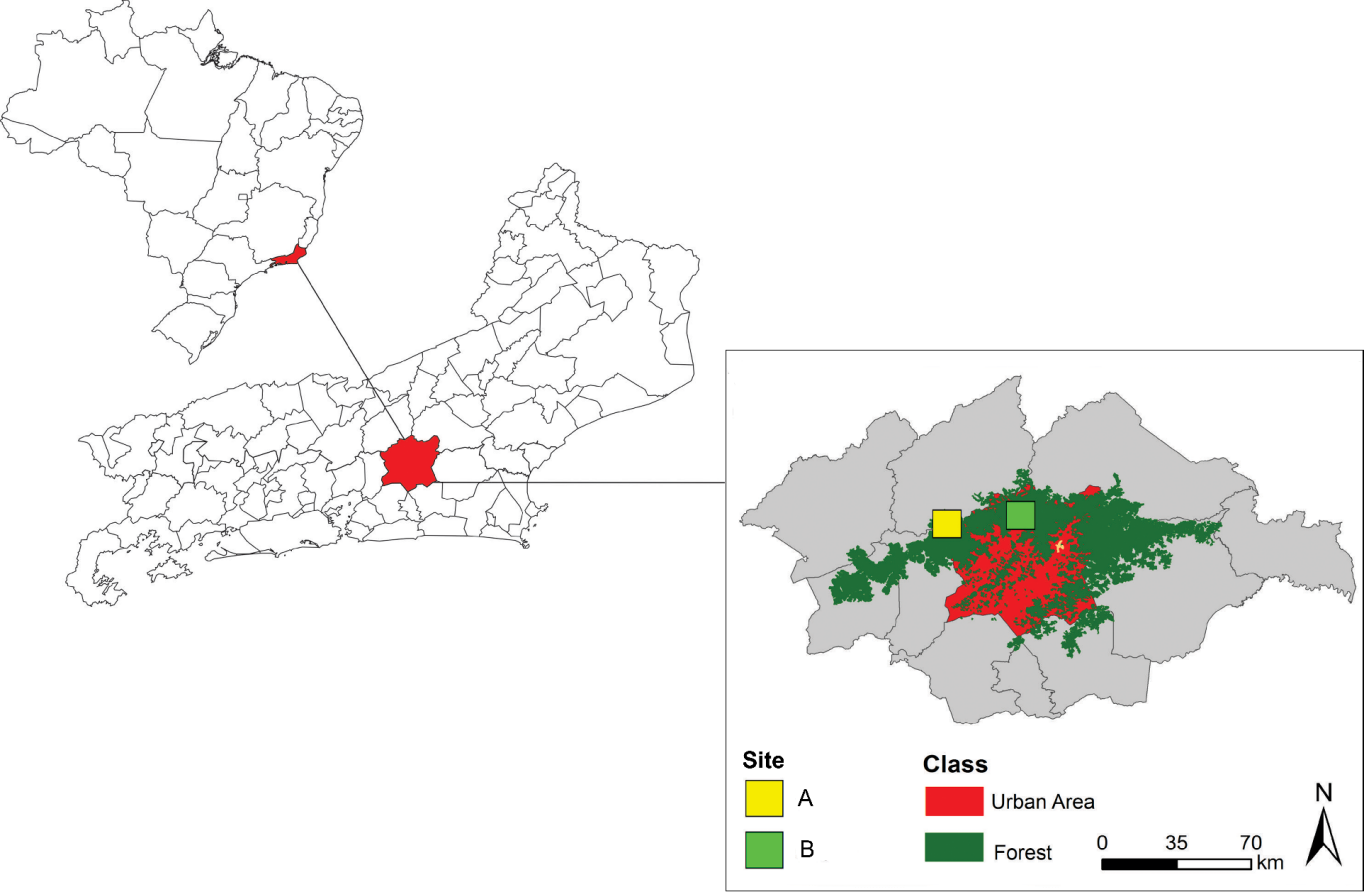
Location of study area and sampling sites in the Guapiaçu Ecological Reserve (REGUA), Rio de Janeiro.

Two sampling sites were established (Fig 1), located 4.44 km apart: Site A—forest next to the administration of the reserve, an area featuring wetlands that were reconstructed in 2005; and Site B—forest with secondary vegetation in different succession stages of development (early, middle, and late stages of natural regeneration), with some patches showing biocenotic floristic composition resembling the original structure. Geographical coordinates were obtained using Garmin GPSmap 60CS GPS. Maps were prepared in Arcview10® and edited in Adobe Photoshop CS5® and CorelDraw X5®.

The presence of immature mosquitoes was investigated in potential natural larval habitats (puddles, wetlands, marshes, brooks, natural rock holes, fruit peels, fallen leaves, bamboo, *Heliconia* spp. plants, tree holes, tabular roots, and bromeliads) and artificial larval habitats (e.g., plastic containers and asbestos tanks) found in each site.

Sampling was performed with the aid of dippers and suction tubes. Water was poured into polyethylene trays; larvae and pupae found were removed with the aid of a fine brush and placed in 250-ml plastic bags (Whirl-Pak Bags®) for transport, each labeled with the location, date, and larval habitat type of collection. In the laboratory, immature Culicidae were screened and transferred to small individual bowls, where they were maintained in water from the collection site and periodically supplemented with distilled water, to allow them to complete their development. Exuviae and larvae that did not complete their development were fixed in 70% glacial ethanol and mounted on a microscope glass slide with cover slips in Canada balsam for species identification.

Species identification was performed by direct observation of the morphological characters using a stereomicroscope (ZEISS Stemi SV6®), and were based on Lane (1953a,b) [18,19], Faran & Linthicum (1981) [20], Consoli & Lourenço-de-Oliveira (1994) [21] and Forattini (2002) [7]. For species of the Aedini tribe, the more traditional classification proposed by Wilkerson et al. (2015) [9] was followed, as opposed to the phylogenetic study by Reinert et al. (2009) [22]. Specimens were deposited in the Entomological Collection of the Oswaldo Cruz Institute, under the title “Coleção Mata Atlântica, REGUA” (Atlantic Forest Collection, REGUA).

### Data analyses

A species accumulation curve was generated for each site to evaluate sampling adequacy and to compare species richness between sites, using individual-based interpolation (rarefaction) from reference samples (total number of individuals collected at each site) using the multinomial model (S(est)) (Colwell et al. 2012) [23] in EstimateS software (Colwell 2013 [24]. Because the performance of richness estimators may vary among data and cases (Gotelli and Chao 2013) [25], Chao1-bc (a bias-corrected form of Chao1) and ACE (a non-parametric abundance-based coverage estimator) were also estimated using SPADE software (Chao and Shen 2010) [26]. To characterize the degree of heterogeneity among species detection probabilities for rare species (defined as those with less than 10 individuals), the squared coefficient of variation of species abundance (CV) was estimated (Chao and Shen 2003) [27]. The number of species expected if sample size was increased and the number of species common to both sites were estimated using sample coverage in SPADE software (200 bootstrap replications used to obtain the standard error estimate).

The real dominance coefficient (RDC) was used to measure the dominance pattern among the species in a given habitat, in relation to the whole community analyzed. The RDC was calculated in accordance with Serra-Freire (2002) [28] as RDC = (∑x_i_ / ∑t_i_) · 100, where ∑x_i_ = sum of individuals of a given species and ∑t_i_ = sum of individuals of all the species collected in a given habitat. A similar approach was followed to estimate dominance of the different larval habitats.

To assess species compositional similarity between larval habitats from the two sites, the abundance Morisita–Horn index of Chao et al. (2006) [29] was estimated using SPADE software (Chao et al. 2010) [26], which is a probability-based index that reduces undersampling bias by estimating and compensating for the effects of unseen, shared species. This index has the advantage that it is not strongly sensitive to species richness and sample sizes and is less likely to be dominated by particular species.

To explore associations of species with larval habitats and sites, a principal components analysis was carried out. Data were previously transformed to ln (n+1), and rock hole data were excluded because they were represented by only one specimen.

## Results

From February 2012 to March 2014, 2697 immature organisms were collected from two areas of REGUA, belonging to 56 species of Culicidae S1 Table. A higher number of species was collected from site A (45) than from site B (38 species). At Site A, the five most dominant species were *Psorophora cingulata* (Fabricius, 1805) (20% of total specimens), *Culex corniger* Theobald (12%), *Limatus durhamii* Theobald, 1901 (12%), *Culex usquatus* Dyar, 1918 (10%), and *Culex pleuristriatus* Theobald, 1903 (7%). At Site B, *Cx*. *pleuristriatus* (22%), *Cx*. *usquatus* (20%), *Li*. *durhamii* (10%), *Culex lanei* Oliveira Coutinho & Forattini, 1962 (10%), and *Culex retrosus* Lane & Whitman, 1951 (8%) were the most dominant (Table 1).

**Table 1:** Total numbers of immature mosquito specimens collected from February 2012 to April 2014, at two sites in the Guapiaçu Ecological Reserve (REGUA), Rio de Janeiro. Sites from where a species was collected is shown as A, B, or AB following numbers of specimens. Species with fewer than 10 specimens were excluded from the Table^a^.

Species	A	B	C	D	E	F	G	H	I	J	K	L
*Ae*. *albopictus* (Skuse 1895)	11^A^				1^A^							
*An*. *evansae* (Brethes 1926)		18^AB^		18^B^					2^B^			
*Cq*. *juxtamansonia* (Chagas 1907)			11^A^									
*Cx*. *abnormalis* Lane 1936							18^A^					
*Cx*. *usquatus* Dyar 1918	12^AB^	326^AB^		6^B^	9^AB^				4^B^	15^B^	30^B^	
*Cx*. *lanei* Oliveira Coutinho & Forattini 1962	8^AB^		4^A^		1^B^		28^B^		101^B^			
*Cx*. *pleuristriatus* Theobald 1903	2^B^				342^AB^	6^B^	13^AB^	3^B^	19^B^			
*Cx*. sp.					9^AB^				1^B^		1^B^	
*Cx*. *corniger* Theobald 1903	158^A^											
*Cx*. *mollis* Dyar & Knab 1906	75^A^											
*Cx*. *hedys* Root 1927					28^B^							
*Cx*. *imitator* Theobald 1903					21^AB^							
*Cx*. *retrosus* Lane & Whitman 1951					157^AB^							
*Cx*. *bastagarius* Dyar & Knab 1906	34^B^	7^AB^			6^A^							
*Cx*. *aureus* Lane & Whitman 1951					12^AB^							
*Li*.* durhamii* Theobald 1901	230^AB^	5^B^			43^AB^		14^A^		3^B^			
*Li*.* flavisetosus* de Oliveira Castro 1935	12^B^				8^B^		24^A^	6^B^				
*Li*.* pseudomethysticus* (Bonne-Wepster & Bonne 1920)							17^A^					
*Ps*. *cingulata* (Fabricius 1805)	49^B^	265^A^										
*Ps*. *ferox* (Von Humboldt 1819)		16^A^										
*Ur*. *pulcherrima* Lynch Arribalzaga 1981		9^AB^	50^A^						1^B^			1^B^
*Wy*. *bonnei* (Lane & Cerqueira 1942)					10^AB^							
*Wy*.* edwardsi* (Lane & Cerqueira 1942)	1^B^				139^AB^				2^B^		1^B^	
*Wy*.* longirostris* Theobald 1901					35^AB^							
*Wy*.* quasilongirostris* (Theobald 1907)					22^A^							
*Wy*.* theobaldi* (Lane & Cerqueira 1942)					34^AB^							
*Wy*.* medioalbipes* Lutz 1904					65^AB^				5^B^	5^AB^		
Total number of species (richness)	14	17	5	3	29	2	6	2	11	3	4	1

A = Artificial; B = Depression on ground; C = Wetland; D = Brook; E = Bromeliad tanks; F = Bamboo internodes; G = Fruit peel; H = Fallen leaves; I = Heliconia; J = Tree-hole; K = Roots; L = Rock hole. *Ae*. *= Aedes*, *An*. *= Anopheles*, *Cq*. *= Coquillettidia*, *Cx*. *= Culex*, *Li*. *= Limatus*, *Ps*. *= Psorophora*, *Tx*. *= Toxorynchites*, *Ur*. *= Uranotaenia*, *Wy*. *= Wyeomyia*

^a^Species with fewer than 10 specimens, listed by larval habitat (sites found in parenthesis): Bamboo: *Tr*. *pallidiventer* (Lutz, 1905) (B); wetland: *Cq*. *fasciolata* (Lynch Arribalzaga, 1891) (A), *Cq*. *venezuelensis* (Theobald, 1912) (A); Tree hole: *Cx*. *urichii* (Coquillett, 1906) (B); Tabular roots: *Ae*. *terrens* (Walker, 1856) (B); *Heliconia*: *Wy*. *arthrostigma* (Lutz, 1905) (B), *Wy*. *airosai* Lane & Cerqueira, 1942 (B); Brook: *An*. *albitarsis* Lynch Arribalzaga, 1878 (A); Ground depression: *An*. *eiseni* Coquillett, 1902 (B), *Ae*. *rhyacophilus* (da Costa Lima, 1933) (A), *Ae*. *scapularis* (Rondani, 1848) (AB), *Ae*. *serratus* (Theobald, 1901) (AB), *An*. *albitarsis* (A), *Cx*. *(Aedinus)* Lutz, 1904 (A), *Cx*. *atratus* group Theobald, 1901 (A), *Cx*. *declarator* Dyar and Knab, 1906 (B), *Cx*. *ocossa* Dyar and Knab, 1919 (A), *Ps*. *albipes* (Theobald, 1907) (A); artificial container: *Cx*. *declarator* (B), *Cx*. *ocellatus* Theobald, 1903 (AB), *Cx*. *quinquefasciatus* Say, 1823 (B), *Ur*. *geometrica* Theobald, 1901 (B), Bromeliad: *An*. *cruzii* Dyar & Knab, 1908 (AB), *Tx*. *trichopygus* (Weidemann, 1828) (B), *Wy*. *aningae* Motta & Lourenço-De-Oliveira, 2005 (B), *Wy*. *bourrouli* (Lutz, 1905) (A), *Wy*. *davisi* (Lane & Cerqueira, 1942) (A), *Wy*. *flabellata* (Lane & Cerqueira 1942) (A), *Wy*. *luteoventralis* Theobald, 1901 (A), *Wy*. *pilicauda* Root, 1928 (AB), *Tx*. *purpureus* (Theobald, 1901) (A), *Tx*. *mariae* (Bourroul, 1904) (AB).

**Fig 2:**
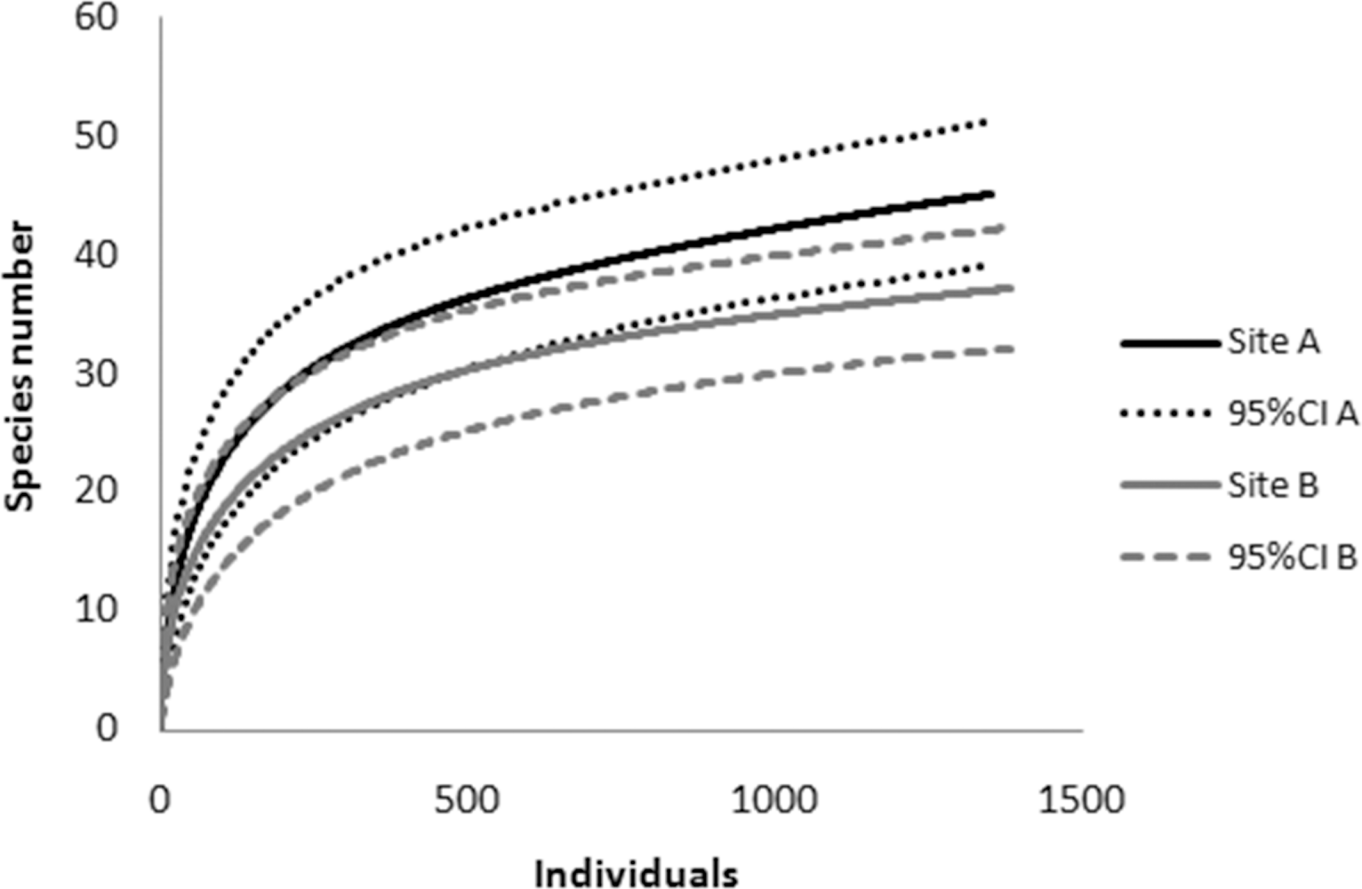
Individual-based interpolation (rarefaction; solid lines) of the reference samples from the two sampling sites (black lines = Site A; gray lines = Site B) in the Guapiaçu Ecological Reserve (REGUA) from a multinomial model, with 95% unconditional confidence intervals (dotted or dashed lines) (based on Colwell et al. 2012).

Species rarefaction curves are shown in Fig 2. At Site A, 45 species were observed; expected species richness based on Chao1-bc was 54 (95% confidence interval (CI) was 47 to 83), and based on ACE was also 54 species (CI 47.8 to 75.9). Using the method of Shen et al. (2002) [30], it was predicted that 7 new species would be discovered in a further survey of 100 individuals, with the 95% confidence interval ranging from 0.1 to 1.3. At Site B, 38 species were detected; Chao1-bc estimated 43 (39.0 to 65.6) and ACE also 43 (39.2, 57.5) species. Less than one (0.5) additional species was predicted in a further survey of 100 individuals (ranging from 0.0 to 1.0). Sampling efficiency was estimated to be 0.99 for each site. Taken together, the results of the present study indicate that the samples obtained provide adequate representation of the species diversity at both sites.

A 16.9% overlap in the 95% confidence intervals was observed around the species accumulation curves between sites (Fig 2). Thus, following the conservative overlap criterion proposed by Colwell et al. (2012) [24], it was inferred that overall species richness did not differ significantly between sites.

In the study area, 140 mosquito larval habitats were identified, two types being artificial (plastic containers and asbestos tanks), and 11 natural habitats in varying number, ranging from ground water (such as wetlands, puddles, and brooks), to phytotelmata (tree holes, bromeliads, bamboo, *Heliconia* plants, and fruit peels) and other microhabitats (such as rock holes) (Table 2). The chi-square test indicated that bromeliads followed by depressions on the ground formed significantly more frequent mosquito larval habitats than the other habitats. Table 3 summarizes the number of specimens, species diversity, and dominant mosquito species in each of the 12 types of larval habitats sampled. Bromeliads presented the highest number of species (52% of all observed species), followed by *Heliconia* (20%), and plastic containers (20%), while the remaining habitats had fewer and similar numbers of species. In all, artificial larval habitats were very productive in terms both of number of species detected (14 species) and number of specimens collected (22% of total sample). *Culex pleuristriatus* was the dominant species in Bromeliads, *Cx*. *usquatus* was dominant in ground depressions, *Cx*. *lanei* in *Heliconia*, and *Li*. *durhamii* in plastic containers. However, when considering all the water collections studied comprising 12 different environments, *Cx*. *usquatus* colonized a larger number of habitat types, being considered dominant in REGUA.

**Table 2:** Larval habitats of Culicidae surveyed from February 2012 to April 2014, at two sites in the Guapiaçu Ecological Reserve (REGUA), Rio de Janeiro.

Larval habitat		Number	Dominance Coefficient (%)*
Artificial	Plastic container	5	3.6^ac^
	Asbestos tank	3	2.1^ac^
Natural	Depression on ground	21	15.0^b^
	Wetland	8	5.7^c^
	Brook	6	4.3^ac^
	Bromeliad	74	52.9^d^
	Bamboo	2	1.4^a^
	Fruit peels	2	1.4^a^
	Fallen leaves	1	0.7^a^
	*Heliconia*	8	5.7^c^
	Tree hole	5	3.6^ac^
	Tabular root	4	2.9^ac^
	Rock hole	1	0.7^a^
Total		140	100

* Exponents with the same letters in the same column indicate no significant difference (p > 0.05); when the letters are not the same, it indicates a significant difference (p < 0.05).

**Table 3:** Diversity of mosquito species found at each of the larval habitat types surveyed from February 2012 to April 2014, at two sites in the Guapiaçu Ecological Reserve (REGUA), Cachoeiras de Macaçu, Rio de Janeiro.

Larval habitat		Number of specimens	Number of species	Dominant species (coefficient, %)
Artificial	Plastic container	221	11	*Li*. *durhamii* (50)
	Asbestos tile	377	6	*Cx*. *corniger* (42)
Natural	Depression on ground	682	17	*Cx*. *usquatus* (48)
	Wetland	80	5	*Ur*. *pulcherrima* (63)
	Brook	28	3	*An*. *evansae* (64)
	Bromeliad	972	29	*Cx*. *pleuristriatus* (35)
	Bamboo	9	2	*Cx*. *pleuristriatus* (67)
	Fruit peels	114	6	*Cx*. *lanei* (25)
	Fallen leaves	9	2	*Li*. *flavisetosus* (67)
	*Heliconia*	141	11	*Cx*. *lanei* (72)
	Tree hole	25	3	*Cx*. *usquatus* (60)
	Roots	38	4	*Cx*. *usquatus* (79)
	Rock hole	1	1	*Ur*. *pulcherrima* (100)
Total		2697	56	*Cx*. *usquatus* (15)

An. = Anopheles; Cx. = Culex; Li. = Limatus; Ur. = Uranotaenia.

Sites A and B shared an observed 25 species out of 56, close to the true number of shared species estimated to be 26 (95% CI 25 to 39). The type of larval habitats differed between sites (Chi-square test, p < 0.001). At Site A, there were 7 types: wetland, tree holes, fallen leaves, fruit peels, ground depressions, artificial container (asbestos tile) and bromeliads. At Site B, only wetlands were missing. Considering only habitat types common to both sites (tree holes, fallen leaves, fruit peels, depression on the ground, artificial containers and bromeliads), 48% of species were shared between sites out of 50 total species found in these habitats; excluding rare species (those with 10 specimens or less), 69% species were shared between sites.

**Fig 3:**
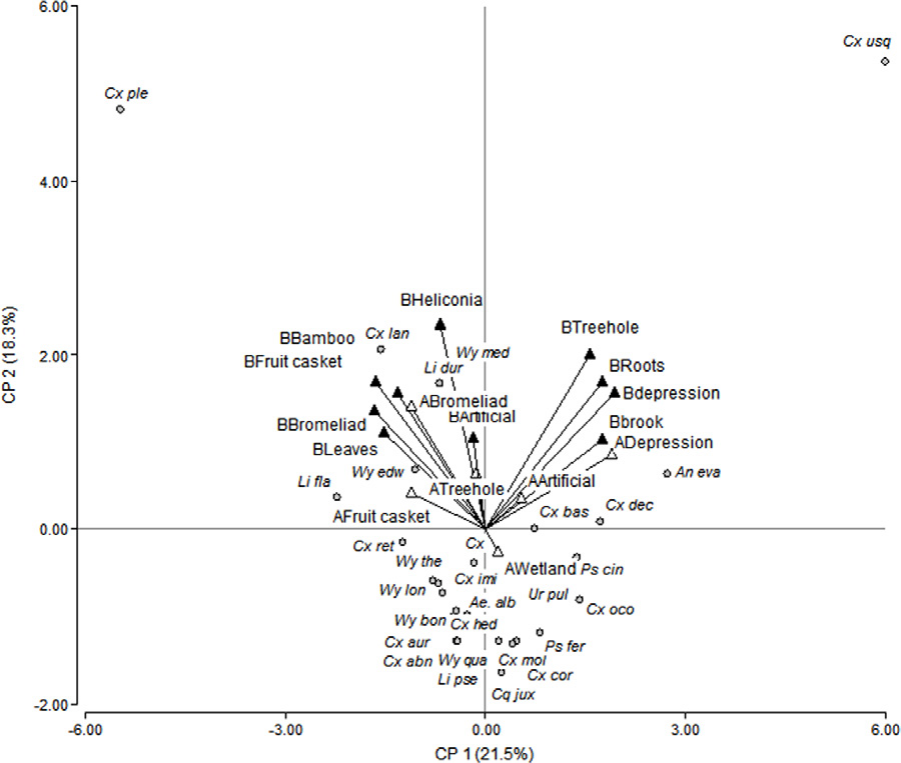
Principal components analysis biplot of the mosquito species found in the larval habitats surveyed at each of the two study sites (Site A in white and Site B in black triangles) in the Guapiaçu Ecological Reserve (REGUA). Species represented by less than 10 specimens (<5% of the sample) were excluded.

Fig 3 shows a biplot resulting from a principal components analysis of the species found in the larval habitats surveyed at each of the two study sites. For this analysis, species represented by less than 10 specimens (less than 5% of the total sample) were excluded to simplify visualization (including all species led to similar clustering). The first two components explained 40% of the data variability. The first component sorted mostly ground water larval habitats to the right side of the graph from phytotelmata to the left. The second component separated temporary ground water on the top right from more permanent wetlands on the bottom right of the plot. The inclusion of tree holes and roots from Site B on the right cluster may be explained by the presence of *Cx*. *usquatus*. *Cx*. *pleuristriatus* and species of Sabethini were more closely associated with phytotelmata (see also Table 1); *Li*. *durhamii* was associated with artificial (plastic) containers, while *Cx*. *usquatus*, *Ur*. *pulcherrima*, *An*. *evansae*, *Ps*. *cingulata*, and some *Culex* species were associated with the ground water habitats. Other clusters of species worth mentioning, although collected in low numbers, include *Coquillettidia* species, which were found exclusively in wetlands (Fig 3 and Table 1), and *Toxorynchites*, found in bromeliads (Table 1).

Table 4 presents a similarity analysis of species composition of larval habitat found at the two sites (excluding habitats with fewer than 25 individuals). Artificial habitats, ground depressions, and fruit peels showed moderate to low similarity based on species presence and their relative abundances. In contrast, the community of mosquitoes found in bromeliads was comparable between sites, with 0.79 similarity values. Bromeliads were larval habitats of 28 Culicidae species, 16 common between the two collection sites, five species were only found in Site B (*Cx*. *lanei*, *Cx*. *hedys*, *Li*. *flavisetosus*, *Tx*. *trichopygus*, and *Wy*. *aningae)*, and eight were found only in Site A (*Ae*. *albopictus*, *Cx*. *bastagarius*, *Tx*. *purpureus*, *Wy*. *bourrouli*, *Wy*. *davisi*, *Wy*. *flabellata*, *Wy*. *luteoventralis*, and *Wy*. *quasilongirostris*) (Table 1).

**Table 4:** Species composition similarity between larval habitats surveyed from February 2012 to April 2014, at two sites in the Guapiaçu Ecological Reserve (REGUA), Cachoeiras de Macaçu, Rio de Janeiro.

Larval habitat	Observed individuals		Observed species			Abundance
	A	B	A	B	Shared	Similarity*
Artificial	377	221	6	11	3	0.49 ± 0.04
Depression	431	225	14	4	2	0.38 ± 0.04
Bromeliad	384	588	24	21	16	0.79 ± 0.04
Fruit peels	78	36	5	2	1	0.03 ± 0.02

*Adjusted estimator ± standard error of 200 bootstrap replications (Chao et al. 2012).

## Discussion

In the study area, 56 mosquito species were identified, a similar value to richness estimates from the same area based on adult mosquito collections (Alencar et al. 2015) [15] and from other Atlantic Forest sites (e.g. Guimarães et al. 1989) [31]. However, species composition partly differed between studies. Within a similar time frame (February 2012—January 2014), 42% of the species had been collected at REGUA also as adults (Alencar et al. 2015) [15], *Limatus paraensis* was detected from ovitraps (Alencar et al. 2016), 33 species were collected as larvae but not as adults, and a further 24 species were not found as larvae in the current study, bringing the total number of species to 82 S1 Appendix.

The most frequently captured (larval) species were *Cx*. *usquatus*, found in most habitat types but more common in temporary ground depressions, and *Cx*. *pleuristriatus*, collected mostly from bromeliads and also other phytotelmata and artificial containers. Other frequent species were *Ps*. *cingulata*, *Li*. *durhamii*, and *Cx*. *corniger*. *Mansonia titillans*, an abundant species in previous adult collections in REGUA (Alencar et al. 2015) [15], and other *Mansonia* species were not detected in the present survey. Species belonging to the genera *Haemagogus* Williston, 1896, *Sabethes* Robineau-Desvoidy, 1827 and *Runchomyia* Theobald 1903, found in the region (Alencar et al. 2015, 2016) [15] were not detected either, which may be related to the number of tree holes and bamboo internodes examined. Height of sampling may have played a small role, as habitats were mostly examined found from ground level up to 2 m. However, even though *Haemagogus* activity is linked to the tree canopy Forattini (1965), Alencar et al. (2016) found *Haemagogus leucocelaenus* colonizing ovitraps installed at all heights from ground level and up to 9 m above the ground at REGUA. On the other hand, a larger proportion of *Cx (Mcx*.*)* and *Wyeomyia* species, frequent inhabitants of bromeliads, were collected compared to the adult surveys of Alencar et al. (2015) [15].

Among the species collected, some are known or suspected vectors of pathogens to humans and/or veterinary relevance. For example, *Aedes scapularis* transmits the dog heartworm *Dirofilaria immitis* Leidy (Labarthe et al. 1998). It is a likely vector of Mayaro and Ilheus virus based on vector competence tests (Aitken & Anderson 1959) and field isolations (Pauvolid-Corrêa et al. 2013), and a suspected vector of Yellow fever in Colombia, besides VEEV, Kairi, Melao, Cache Valley, among other viruses (Forattini 2002 [7]). Together with *Ps*. *ferox*, they have been incriminated as vectors of Rocio virus (Mitchell et al. 1984, Mitchell and Forattini 1986) and are probably involved in orbivirus transmission to horses in Peru (Méndez-López et al. 2015). *Anopheles evansae* may play a secondary role in malaria transmission in neotropical areas where it may be found in high densities (Xavier and Rebêlo 1999). *Aedes albopictus* is a good vector of dengue (Rezza 2012; Marcondes and Ximenes, 2016) and Zika among other arbovirus (Grard et al. 2014), and may be intermediate for yellow fever between sylvatic and urban areas in Brazil (Gomes et al. 1999). This exotic mosquito is a common inhabitant of artificial containers and tree holes mostly in suburban and rural areas, unlike *Ae*. *aegypti*, which is far more urban and closely associated to urbanization (Vezzani and Carbajo 2008, Brady et al. 2014), a fact that could explain why the latter species was not found in artificial containers during the study (Correa et al 2014; Mangudo et al. 2015).

The presence of *Ae*. *albopictus* and other species susceptible to flavivirus infection and transmission may pose a risk also for the spread of recently newcomer viruses: Zika virus, first isolated in 1947 in Africa, has rapidly spread throughout the Americas since its introduction in Brazil in 2015 (Vorou 2016, Petersen et al 2016). Although mostly linked to urban/suburban environments due to *Ae*. *aegypti* and *Ae*. *albopictus* being its main vectors, little is known about the vector capacity of American mosquitoes. There is growing concern about the possibility of Zika virus establishing sylvatic transmission cycles because of the high diversity of potential vectors, enhanced by the recent detection of the virus in neotropical primates (Favoretto et al. 2016; Althouse et al 2016). The presence of *Aedes albopictus* and other arbovirus vectors in REGUA highlight the need to survey arboviral activity in the area.

Similarity analysis of species composition indicated that the larval mosquito community found in artificial habitats, temporary ground water and fruit peels differed between sites, while bromeliads mosquito fauna were almost 80% similar. Differences between fruit peel samples may be an artifact due to low number of samples, however, differences in larval community between sites in ground water may be related to differing local habitat conditions because site A was more open, with presence of wetland and site B had higher canopy cover and more conserved forest conditions. A principal components analysis showed that species segregation was more evident between larval habitats than between sites, although some species were found only in one site, as will be discussed in following paragraphs.

Depressions on the ground form temporary rain pools that, depending on rainfall and temperature conditions in the region, may only last for a few days; thus, mosquitoes that develop quickly can exploit these habitats, such as *Ae*. (*Ochlerotatus*) *scapularis* and some *Culex* and *Psorophora* mosquitoes as those found in this study. *Ps*. *cingulata* was a dominant species in depressions on the ground, while *Cx*. *usquatus* colonized a higher number of larval habitats, being frequently dominant in REGUA. In the present study, *Psorophora albipes* (Theobald, 1907) and *Ps*. *ferox* were collected exclusively in ground water, and mostly in temporary waters; *Ps*. *cingulata* was also collected from artificial containers. These species lay eggs that can tolerate drought conditions and may remain viable for several years or until the next flood event (Foss & Deyrup 2007) [32], a strategy that would allow them to colonize recently formed ground pools and that is common to all species of *Psorophora* in general [7]. According to Belkin et al. (1971) [33], larvae of these species develop in rain pools of forested areas, an observation corroborated by Consoli and Lourenço-De-Oliveira (1994) [34], specifically addressing the larvae of subgenus *Janthinosoma* Lutz, 1908 which includes *Ps*. *albipes* and *Ps*. *cingulata*. They reaffirmed that larvae develop in shallow and temporary pools in the ground, characterized by the abundance of emergent vegetation or by shade afforded by the tree canopy.

Studies by Alencar et al. (2010) [35] in areas of the Serra do Mar State Park showed that *Li*. *durhamii* was collected exclusively from human items, such as rubber boots, abandoned in the park. Guimarães et al. (1985) [36] reported that among Sabethini studied in the Serra dos Órgãos National Park, *Li*. *durhamii* was among the species best adapted to the urban environment, with a high potential for domiciliation and adaptation to different types of artificial larval habitats. Lourenço-de-Oliveira et al. (1986) [37] found immature *Li*. *durhamii* exclusively in artificial containers in peri-urban areas of the city of Rio de Janeiro. Consistently, in REGUA, 78% of *Li*. *durhamii* larvae were collected from artificial containers, but the species was also developing in fruit peels, *Heliconia* bracts, bromeliad tanks, and a few specimens were found in temporary ground pools.

*Culex usquatus* was the species that colonized the highest number of larval habitats, and emerged as the dominant Culicidae species in the REGUA area. Alencar et al. (2010) [35] also observed *Culex* species colonizing a variety of larval habitats and representing the second highest rate of dominance during the study period in the Serra do Mar State Park, which stretches from the border of Rio de Janeiro to Itariri in the southern part of São Paulo, Brazil. Even though the involvement of *Cx*. *usquatus* as a vector of pathogens to humans is currently unknown, *Culex* (*Culex*) mosquitoes are known vectors of Saint Louis encephalitis (SLEV) and West Nile virus (Diaz et al. 2013), and SLEV has been isolated from species of the same complex, *Cx*. *coronator* (Vasconcelos et al. 1991).

At Site A, there was a predominance of species of the Mansoniini tribe, whose immature fauna fix to aquatic vegetation tissues (Forattini, 2002) [7]. This flooded area of the reserve was covered with aquatic plants, facilitating the proliferation of these species, which are usually very aggressive, with large populations (Consoli & Lourenço-de-Oliveira, 1994) [21].

Unlike Site A, most of the Sabethini collected concentrated in Site B. These mosquitoes are essentially tropical and their immature forms develop in phytotelmata; thus, their larval habitats are natural plant containers found in wild environments [7, 19, 38] (Lane, 1953b; Forattini, 1965b; and Forattini, 2002), which explains their high representation in Site B, the sample area that has the highest degree of environmental preservation.

Negligible correlations between species diversity and the abiotic environmental variables measured during the sampling period (temperature and pH) suggest that in REGUA these variables did not act as limiting factors for the mosquito species studied.

Few immature *An*. *cruzii* Dyar & Knab, 1908 were collected, and they were found exclusively colonizing bromeliads. This species has frequently been incriminated in the transmission of the etiologic agent of human and simian malaria in areas with Atlantic Forest vegetation, on the coast of South and Southeast Brazil (Deane 1986) [39]. The low number of anophelines of the subgenus *Kerteszia* Theobald, 1905 in the Atlantic Forest has also been reported by Aragão (1956) [40]. These authors observed that significant levels of rainfall are not related to the number of precipitation events, but to the type of precipitation. Periods of more prolonged rains favor the proliferation of bromeliads, and in periods of drought, with more rare and scattered showers, these plants, being practically the only known larval habitat of this subgenus, sustain the proliferation of the mosquitoes.

Regarding the larval habitat of *Wy*. *bonnei* (Lane & Cerqueira, 1942), *Wy*. *edwardsi* (Lane & Cerqueira 1942), *Wy*. *flabellata* (Lane & Cerqueira, 1942), *Wy*. *longirostris* Theobald, 1901, *Wy*. *pilicauda* Root, 1928, *Wy*. *quasilongirostris* (Theobald, 1907), and *Wy*. *theobaldi* (Lane & Cerqueira, 1942), at least with regard to REGUA, the presence of immature forms was noted only in bromeliads, suggesting no variability in the choice of oviposition sites. It is important to note the fact that the species *Wy*. *quasilongirostris*, *Wy*. *flabellata*, and *Wy*. *davisi* (Lane & Cerqueira, 1942) were only found in Site A. Silva et al. (2007) [41] suggest that bromeliad communities, because they are small, relatively simple and abundant, offer unparalleled advantages for studies of ecological processes in tropical areas such as research on community structure, colonization, dispersal, and other ecological aspects. It is worth mentioning that most of *Wyeomyia* species are Neotropical and as far as it is known, they breed in the water accumulated on Bromeliaceae and Araceae leaves [7].

*Aedes terrens* (Walker, 1856) was only detected in the water collected from tabular roots. This finding corroborates the observations made by Lozovei & Luz (1976) [42] and Neves & Faria (1977) [43], although it was often collected in artificial habitats in Paraná by Lopes (1997, 2002) [44, 45] and in Minas Gerais by Alencar et al. (2013) [46].

*Trichoprosopon pallidiventer* (Lutz, 1905) was collected from bamboo internodes. According to Forattini & Marques (2000) [47], species of the genus *Trichoprosopon* Theobald, 1901 are sylvatic mosquitoes frequently collected from phytotelmata, such as tree holes, leaf axils, and fruit peels.

The artificial larval habitats found in REGUA were colonized by a great diversity of species and high abundance as well. Considering that both natural and artificial containers in REGUA were similarly colonized by mosquitoes, human artifacts left by the public in the area that collect water may promote an increase in mosquito populations.

## Supporting Information

S1 Appendix Mosquito species detected in REGUA between January 2012 and March 2014. Species presence is based on surveys of larval habitats (this work) adult collections (Alencar et al. 2015) and ovitrap collections (Alencar et al. 2016).
(XLS)

S1 Table Immature specimens per sampling site. Numbers of specimens collected per species between January 2012 and March 2014 from each larval habitat type at two sampling sites (Site A and Site B).
(XLS)
